# *In vitro* effectivity of three approved drugs and their synergistic interaction against *Leishmania infantum*

**DOI:** 10.7705/biomedica.4891

**Published:** 2020-08-20

**Authors:** Iman Fathy Abou-El-Naga, Rasha Fadly Mady, Nermine Mogahed Fawzy Hussien Mogahed

**Affiliations:** 1 Medical Parasitology Department, Faculty of Medicine, Alexandria University, Alexandria, Egypt Alexandria University Medical Parasitology Department Faculty of Medicine Alexandria University Alexandria Egypt

**Keywords:** Leishmania infantum, drug synergism, apoptosis, autophagy, Leishmania infantum, sinergismo farmacológico, apoptosis, autofagia

## Abstract

**Introduction:**

Leishmaniasis remains one of the neglected tropical diseases. Repurposing existing drugs has proven to be successful for treating neglected tropical diseases while combination therapy is a strategic alternative for the treatment of infectious diseases.

Auranofin, lopinavir/ritonavir, and sorafenib are FDA approved drugs used in the treatment of diverse diseases by acting on different essential biological enzymes.

**Objective:**

To evaluate the effects of monotherapy and combined therapies with the three drugs against *Leishmania infantum*.

**Materials and methods:**

We compared the leishmanicidal effects of the three drugs on promastigotes *in vitro* as regards the parasite count, the drug concentration providing a half-maximal response, and the ultrastructural changes of the parasite. We determined the fractional inhibitory concentration index of combined drugs in two ways, as well as the activity of the three drugs together to establish their synergetic effect.

**Results:**

The monotherapy with the three drugs was effective with auranofin showing the best leishmanicidal effect (EC50=1.5 µM), whereas sorafinib reduced parasite growth at EC50=2.5 µM. The scanning electron microscopy of promastigotes from all treated media showed distortion in the shape with loss of flagella and bleb formation. Acidocalcinosis was evident by transmission electron microscopy with all treatments suggesting apoptosis.

Treatment with lopinavir/ritonavir showed signs of autophagy. The two-way combination of the drugs led to additive interactions while the combination of the three drugs showed synergistic action.

**Conclusion:**

Each drug when used as monotherapy against *Leishmania* spp. was effective, but the combination therapy was more effective than the individual drugs due to the additive or synergistic effects.

## Resumen

**Introducción.:**

La leishmaniasis sigue siendo una de las enfermedades tropicales desatendidas. La reutilización de medicamentos existentes ha demostrado ser exitosa para tratar enfermedades tropicales desatendidas y la terapia combinada es una alternativa estratégica para el tratamiento de enfermedades infecciosas. Auranofin, lopinavir/ritonavir y sorafenib son medicamentos aprobados por la *Food and Drug Administration* (FDA) de Estados Unidos utilizados en el tratamiento de diversas enfermedades, pues actúan sobre diferentes enzimas biológicas esenciales.

**Objetivo.:**

Evaluar los efectos terapéuticos de la monoterapia y de los tres fármacos combinados contra *Leishmania infantum*.

**Materiales y métodos.:**

Los efectos leishmanicidas de los tres fármacos sobre los promastigotes se compararon *in vitro* en cuanto al recuento de parásitos, la concentración del fármaco que proporcionara una respuesta semimáxima y los cambios ultraestructurales del parásito. Se calculó el índice de concentración inhibitoria de fracciones de fármacos combinados de dos maneras y la actividad de los tres fármacos juntos para determinar el efecto sinérgico.

**Resultados.:**

La monoterapia con los tres medicamentos fue efectiva, pero la auranofina tuvo el mejor efecto antileishmanicida con un CE50 de 1,5 µM, en tanto que el sorafinib redujo el crecimiento del parásito con un CE50 de 2,5 µM. La microscopía electronica de barrido de promastigotes de todos los medios tratados mostró una distorsión en la forma, con pérdida de flagelos y formación de ampollas. La acidocalcinosis fue evidente por microscopía electrónica de transmisión con todos los tratamientos, lo que sugiere apoptosis. El tratamiento con lopinavir/ritonavir mostró signos de autofagia. La combinación de dos medicamentos condujo a interacciones aditivas, mientras que la combinación de las tres drogas produjo una acción sinérgica.

**Conclusión.:**

Los tres medicamentos usados como monoterapia contra *Leishmania* spp. fueron efectivos, pero el tratamiento combinado lo fue en mayor medida debido a los efectos aditivos o sinérgicos.

Leishmaniasis is one of the neglected tropical diseases. It affects as many as 12 million people living in endemic areas in 98 countries. About 350 million people are considered to be at risk, most of them in developing countries ^1^^-^^3^. *Leishmania* species cause a wide clinical spectrum that includes cutaneous, mucocutaneous, and visceral leishmaniasis. The most common is the cutaneous form, which causes disfiguring and stigmatizing skin lesions whereas mucocutaneous leishmaniasis is significantly less common. Visceral leishmaniasis is fatal if not treated ^4^.

Currently, limited choices of drugs are used for the treatment of leishmaniasis. There are no approved vaccines nor prophylactic drugs. Pentavalent antimonial compounds, sodium stibogluconate, pentamidine, various amphotericin B formulations, miltefosine, and paromomycin are the approved medications at the moment. Imiquimod and sitamaquine are under clinical assessment ^5^. However, the available drugs have limitations which include toxicity, long courses, high costs, undesirable route of administration, teratogenicity, and drug resistance.

So far, no safe and effective anti-*Leishmania* drug is available in the market ^6^. Recent research funded by various organizations is only directed towards clinical trials and diagnostic studies of leishmaniasis in endemic countries. Consequently, there is still an urgent need to develop new therapeutics for leishmaniasis.

New drug trials are presently aimed at interfering with vital biochemical and metabolic pathways of the parasite and in this rationale, enzymes are the most important focus. The target enzymes in the parasite should have major structural and functional differences from those of the mammalian host to achieve selective inhibition of the target sites ^7^.

Repurposing existing drugs has proven to be successful for treating neglected tropical diseases. The new uses approved by the US Food and Drug Administration (FDA) are a shortcut between the preclinical testing and clinical trials. This strategy reduces the funds needed for the preclinical researches and the study of the safety profiles and pharmacological characteristics ^8^^,^^9^.

For this study, we chose three FDA-approved drugs, namely auranofin, lopinavir/ritonavir and sorafenib, which act as inhibitors of different protease enzymes to study their effect as monotherapy and combination therapy on *Leishmania infantum*. *Leishmania* spp. proteases are very important virulence factors as they are involved in host tissue invasion, survival inside macrophages, and host immune response modulation for which they are considered good targets ^10^. The efficacy of the drugs was compared to that of amphotericin B, a polyene antibiotic that acts on the membrane sterols of *Leishmania spp.* promastigotes producing the loss of the permeability barrier to small metabolites ^11^. Although amphotericin B is widely used in the treatment of leishmaniasis, its toxicity is considerable ^12^.

Auranofin is a gold-containing drug used in the treatment of rheumatoid arthritis. It emerged as a strong inhibitor of mammalian thioredoxin reductases ^13^. Recently, the drug showed a remarkable antiparasitic activity by inhibiting those enzymes involved in the control of the reduction/oxidation (redox) process. These enzymes are essential for maintaining intracellular levels of reactive oxygen species. *Leishmania* and other Trypanosomatids contain trypanothione reductase, a key enzyme of redox metabolism ^14^.

Trypanothione reductase and mammalian glutathione reductase show notable differences in the structure validating specific inhibitors designed against trypanothione reductase as an ideal drug against *Leishmania* spp. without changing the mammalian glutathione reductase activity ^7^.

Lopinavir/ritonavir is a highly active anti-retroviral medication used against HIV ^15^. The drug is an aspartyl peptidase inhibitor composed of two anti- retroviral drugs: lopinavir and ritonavir, in a ratio of 4:1 ^16^. Peptidases are essential in a wide range of biological functions ^17^. They are recognized as therapeutic targets for important diseases and many micro-organisms including *Leishmania*^18^^-^^20^. These enzymes are classified into five distinct clans (AA, AC, AD, AE, and AF) and 16 families according to the MEROPS database. The classical aspartic peptidases Clan AA is further subdivided into eight families of which family A2 includes the HIV peptidase. In Trypanosomidae, the aspartic peptidases belong to two clans: clan AA and clan AD ^21^.

Recently, the co-infection of *Leishmania* spp. and HIV has been increasingly reported in leishmaniasis endemic areas ^4^. The introduction of the highly active antiretroviral therapy (HAART) has shown a recognizable decrease in *Leishmania*/HIV co-infection as regards incidence, pathology, and clinical presentation of the disease ^22^. Experimental studies on different *Leishmania* species using HIV peptidase inhibitors have enforced the epidemiological results documenting a decrease in the incidence of *Leishmania*/HIV co-infection after treatment with these drugs ^21^^-^^25^.

Sorafenib is a multi-kinase inhibitor used for the treatment of advanced hepatocellular and renal cell carcinoma. Recently, it was identified as an active agent against *L. donovani* and different species of *Leishmania* causing cutaneous leishmaniasis ^26^. A large number of kinases, especially cyclin- dependent and mitogen-activated kinases, are responsible for cell-cycle control in *Leishmania*. Although kinases are recognized as targets for many diseases, they have been poorly studied as targets for *Leishmania*^27^^).^ The anti-leishmanial potency of sorafenib is due to the non-specific inhibition of many diverse protein kinases rather than that of the mammalian kinases ^28^.

Combination therapy is a strategic alternative for the treatment of infectious diseases. It is currently considered as one of the most rational alternatives to increase drug activity, reduce treatment duration and dosage, reduce toxicity, and delay or prevent drug resistance. It has been efficiently used in the treatment of malaria, tuberculosis, and AIDS ^29^. However, it is uncommon to treat leishmaniasis with combined drugs ^30^^-^^32^, but the need for combination therapy against leishmaniasis has emerged ^33^.

In this study, we evaluated the anti-leishmanial effect of auranofin, lopinavir/ritonavir and sorafenib against *L. infantum* promastigotes compared to the gold standard drug for leishmaniasis, amphotericin B. The synergistic, additive or antagonistic effects of combined therapy were also investigated, as well as the morphological changes of the parasite treated with the aforementioned drugs at the ultrastructural level.

## Materials and methods

### Maintenance of the Leishmania strain

*Leishmania infantum* MON1 is the visceral leishmaniasis strain used in this study. It was kindly provided by Professor Jean Dupouy Camet, president of the European Federation of Parasitologists. It was further maintained in the Laboratory of Medical Parasitology Department, Faculty of Medicin at Alexandria University. *Leishmania infantum* promastigotes were maintained under standard culture conditions in Novey-MacNeal-Nicoll (NNN) media. Parasites were sub-cultured every seven days ^34^.

### Tested drugs

Three commercially available FDA-approved drugs were used in this study: auranofin, purchased from Abbott; lopinavir/ritonavir, purchased from Astellas pharma SPA, and sorafenib, purchased from Bayer. Amphotericin B was used as the gold standard.

### *Determination of the* in vitro *anti-leishmanial activity*

Ten μM stock solutions were prepared from each drug in 1% DMSO. The negative control was prepared from the 1% DMSO and the positive control was 10 μM of AmB. We incubated 1 x 10^6^ *Leishmania* promastigotes suspended in 100 μL of culture media for three hours before adding the test drugs. After 48 hours of incubation with each drug preparation at 25°C, an aliquot of each tube was added to an equal amount of a solution containing 0.2% formalin to stop parasite movement and facilitate the counting using a Neubauer chamber ^35^. Auranofin, lopinavir/ritonavir, and sorafenib were tested in ascending concentrations ranging from 0.1 to 20 μM. Amphotericin B was tested in a dilution range of 0.01 to 17 μM.

### Determination of the fractional inhibitory concentration index, isobologram construction, and classification of the nature of the interaction

The fractional inhibitory concentration (FIC) is the one that caused a 50% decrease in the growth (EC_50_) of promastigotes. It was calculated for each tested drug and each concentration. All tests were performed in triplicate ^9^.

To test for synergy, drugs were evaluated in quadruplicate individually to determine the EC_25_; each compound in a pair was required to inhibit 25 ± 10% of growth in untreated media.

Drug combinations that showed possible synergism were subjected to formal isobologram analysis using the fixed ratio method ^36^.

Serial two-fold dilutions were performed in triplicate. We calculated the EC_50_ for each drug ratio. The fractional inhibitory concentrations were calculated as the following:

EC_50_ when in combination / EC_50_ of individual drug

The sum of the FIC was calculated as follows: Σ FIC = FIC drug A + FIC drug B. The mean sum of the FIC (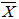 Σ FIC) was calculated as the average of SFIC from the three different fixed ratios. The interactions were considered synergistic for 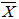 Σ FIC ≤ 0.5, additive for 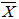 Σ FIC between 0.5 and 4, and antagonistic for 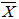 Σ FIC >4 ^9^.

### Ultrastructural study

A scanning electron microscope (SEM) (JEOL-JSM-25 SII™) and a transmission electron microscope (TEM) (JEOL 100 CX™) were used to examine *L. infantum* promastigotes after their treatment with auranofin, lopinavir/ritonavir, and sorafenib for 48 h at 26°C in comparison to positive and negative controls. The specimens were processed for SEM and TEM ^37^^,^^38^.

### Statistical analysis

All parasite burden data were expressed as the mean ± standard deviation. Abnormally distributed data were expressed using the median (min-max) and compared using the Kruskal Wallis test. Significance between groups was determined using the Mann Whitney test. A p-value of <0.05 was considered statistically significant ^39^.

## Results

### In vitro *anti-leishmanial activity*

It was clear that all individual drugs, auranofin, lopinavir/ritonavir, sorafenib, and amphotericin B, limited *in vitro* parasite growth after 48 hours of parasite replication whereas DMSO had no significant effect. Lopinavir/ritonavir reduced parasite growth at 1.7 µM. As regards to auranofin, the lower drug concentration limiting parasite growth by 50% (EC_50_) was 1.5 µM. sorafenib while AmB showed the highest EC_50_ concentrations (2.5 µM and 2 µM, respectively) (tables 1 and 2).

**Table 1:** Effect of different drugs on in vitro proliferation of *Leishmania infantum* promastigotes

Drug	Growth percentage
Auranofin (3 mg)	4.27
Lopinavir/ritonavir (200 mg)	6.8
Sorafenib (200 mg)	9.84
Amphotericin B	11
Auranofin+ lopinavir/ritonavir (combination A)	3.75
Lopinavir/ritonavir + sorafenib (combination B)	5.1
Auranofin + sorafenib (combination C)	8.25
Auranofin + sorafenib+ lopinavir/ritonavir (combination D)	4.75

**Table 2:** EC50* of the different drugs

Drug	EC50 (μM)
Auranofin (3 mg)	1.5
Lopinavir/ritonavir (200 mg)	1.7
Sorafenib (200 mg)	2.5
Amphotericin B	2

* EC_50_ values are means of triplicate assays.

### Synergy testing and isobologram analysis

EC_25_ values were measured for each of the drugs in every possible combination (table 3). Four combinations were tested in formal isobologram analyses to quantify the interactions by this standard method.

**Table 3:** EC_25_* of the different drugs in every combination

Drug	Lopinavir/ritonavir	Auranofin	Sorafenib
Auranofin + lopinavir/ritonavir (combination A)	0.77 μM	2.3 μM	
Lopinavir/ritonavir + sorafenib (combination B)	1 μM		2 μM
Auranofin + sorafenib (combination C)		1.8 μM	1.2 μM
Auranofin + sorafenib + lopinavir/ritonavir (combination D)	0.3 μM	0.3 μM	0.3 μM

EC_25_ values are means of triplicate assays.

### Fractional inhibitory concentration

Sum of FIC for combination A (auranofin + lopinavir/ritonavir) = 1.53 + 0.45 = 1.98 = additive**.**

Sum of FIC for combination B (sorafenib + lopinavir/ritonavir) = 0.59 + 0.8 = 1.39 = additive**.**

Sum of FIC for combination C (auranofin + sorafenib) = 1.2 + 0.48 = 1.68 = additive**.**

Sum of FIC for combination D (auranofin + lopinavir/ritonavir + sorafenib) = 0.17 + 0.12 + 0.2 = 0.49 =synergism.

When these results were statistically analyzed, lopinavir/ritonavir showed no significant difference from other drugs either when used alone or in combination. The growth inhibition in auranofin-treated media was significantly greater than that treated with sorafenib and amphotericin B, but no statistically significant difference was found between it and any combination. Despite the reduction in parasite growth, sorafenib was the least potent drug in comparison to auranofin and combinations B, C, and D.

Combinations B, C, and D showed a significant reduction in the growth of promastigotes compared to amphotericin B and combination A (table 4).

**Table 4:** *In vitro* activity of different drugs and combinations against promastigotes

Parasite count in culture media treated with different drugs	Median (min-max)
Auranofin (300 mg)	38 (27 - 77.5)^b^
Lopinavir/ritonavir (200 mg)	70 (18 - 100)^ab^
Sorafenib (200 mg)	101 (40 - 150)^a^
Amphotericin B	107.5 (88 - 137)^a^
Auranofin + lopinavir/ritonavir (combination A)	37.5 (15 - 60)^b^
Lopinavir/ritonavir+ sorafenib (combination B)	51 (20 - 82)^b^
Auranofin + sorafenib (combination C)	82.5 (45 - 120)^ab^
Auranofin+ sorafenib + lopinavir/ritonavir (combination D)	40 (20 - 90)^b^

Abnormally distributed data was expressed using the median (min-max) and compared using the Kruskal Wallis test. Significance between groups was established using the Mann Whitney test.

Different superscripts are statistically significant.

### Ultrastructural study

**Figure 1:**
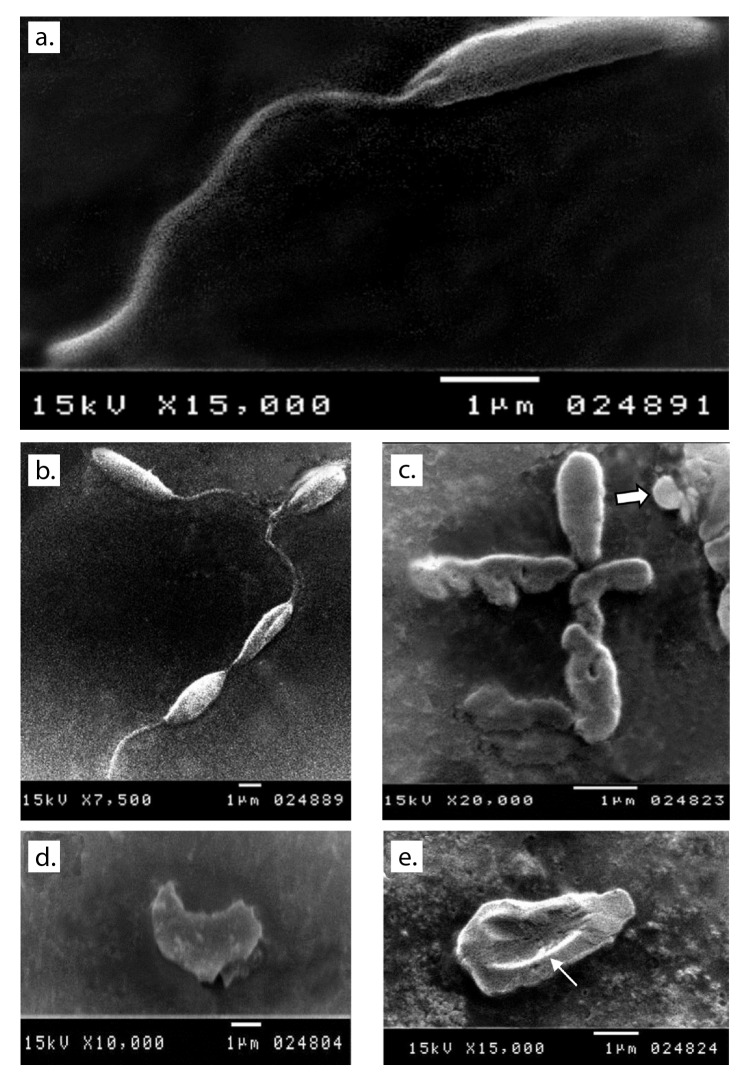
Ultrastructural changes observed in promastigotes from the different media under study. a and b: Normal shape of parasite inoculated in fresh media with no drug added after 48 hours of culture. c: Auranofin-treated parasite showing shape distortion and loss of flagella and some showing a round form (arrow). d: Severe distortion in the shape and loss of flagella with detached membrane in lopinavir/ritonavir-treated promastigote. e: Sorafenib-treated promastigote showing a large dimple (arrow) on its body surface as well as loss of flagella.

Scanning electron microscopy after 48 hours of the culture of parasites inoculated in fresh media with no drug added showed normal morphology (figure 1a and b). Promastigotes from all treated media showed distortion in the parasite shape with loss of flagella and bleb formation. Auranofin-treated promastigotes exhibited severe shape distortion while some showed a rounded form (figure 1c). Irregularities in the cell membrane of the parasite were highly evident in lopinavir/ritonavir-treated promastigotes (figure 1d) while dimple-like structures on the cell surface were observed in sorafenib- treated parasites (figure 1e).

**Figure 2:**
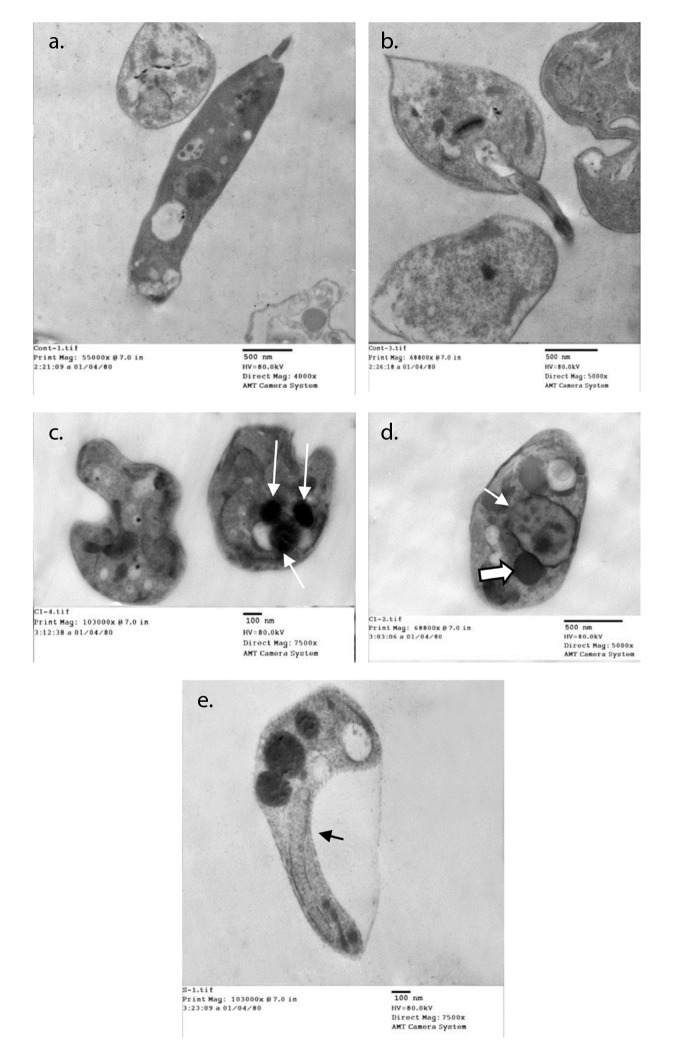
Ultrastructural changes in promastigotes from different media. a and b: Normal parasite inoculated in fresh media with no drug added after 48 hours of culture. c: Auranofin-treated promastigotes showing evident acidocalcinosis (arrows). d: Lopinavir/ritonavir-treated promastigotes showing evident acidocalcinosis (thick arrow) and degenerated nuclear membrane (thin arrow) with condensed chromatin granules close to the nuclear membrane suggesting apoptosis. Vacuoles with different densities and autophagy vesicles with double membrane were also present. e: Sorafenib- treated promastigotes showing acidocalcinosis and shrinkage of the cytoplasm (arrow).

Normal ultrastructure contents were detected using transmission electron microscopy after 48 hours of the culture of parasites inoculated in fresh media with no drug added (figure 2a and b). Acidocalcinosis was evident in the parasites from all treated media, which suggested apoptosis. Auranofin-treated promastigotes showed evident acidocalcinosis (figure 2c). Lopinavir/ritonavir- treated promastigotes showed very evident acidocalcinosis, degenerated nuclear membrane, and chromatin granules suggesting apoptosis. Vacuoles with different densities and autophagy vesicles with double membrane were also present (figure 2d). Sorafenib-induced apoptosis with shrinkage of the cytoplasm (figure 2e) was also evidenced.

## Discussion

Despite several trials, there are no effective vaccines against *Leishmania* spp. at the moment and chemotherapy remains the mainstay for the control of leishmaniasis. The currently used drugs are unsafe, expensive, and lead to resistance ^40^ while the treatment with protease inhibitors has been tried before in several research studies. Accordingly, in this study, we evaluated the effect of three different commercially available enzyme inhibitors against *L. infantum*. We compared the monotherapy and the combination therapy of auranofin, lopinavir/ ritonavir and sorafenib. The drugs were chosen for their well-known history of safe clinical profile and their inhibition of essential enzymes ^41^.

Our results showed that auranofin had the most effective anti-leishmanial activity. It is the only drug that when used individually led to a significant inhibition in the parasite count compared to amphotericin B. At 10 µM, auranofin significantly reduced the parasite count compared to sorafenib but the results were insignificant compared to that of lopinavir/ritonavir. Furthermore, with auranofin, the growth percentage and the LC_50_ of the parasite were the lowest in comparison to lopinavir/ritonavir, sorafenib, and amphotericin B. The effect of auranofin against *L. infantum* is due to the inhibition of trypanothione reductase enzyme, one of the top targets in drug discovery for leishmaniasis, as it protects the parasite from oxidative damage and toxic heavy metals and allows the delivery of the reducing equivalents for DNA synthesis ^14^^,^^42^. Lopinavir/ritonavir was found to have more efficacy than amphotericin B. The proteolytic activity of the HIV protease inhibitors has been demonstrated in other studies of different *Leishmania* species ^21^^,^^24^^,^^25^^,^^43^. Alves, *et al*. ^44^, explained another way of their action through the modulation of innate defense mechanisms via different cellular pathways.

They also showed that, although HIV protease inhibitors are highly efficient to control HIV, they might also influence the course of leishmaniasis in HIV- *Leishmania*-co-infected individuals.

In the present study, sorafenib showed the least leishmanicidal activity among the drugs under study. It did not significantly reduce the promastigotes compared to amphotericin B and had a higher LC_50_. The drug is a multi- kinase inhibitor and was found to be active against *L. donovani* in culture by identifying cycline-dependent and mitogen-activated kinases as targets for anti-leishmanial treatment ^26^^,^^27^. Recently, it was found that sorafenib utilizes a non-apoptotic form of cell death (ferroptosis) on tumor cells. Ferroptosis is a regulated form of cell death resulting from iron-dependent lipid peroxide accumulation as shown by Yu, *et al.*^45^.

Combination therapy with commercially available drugs aims at reducing the costs, toxicity, and duration of the treatment and represents a promising alternative rationale ^46^. Therefore, we tried the drug combinations of two and three compounds. The results showed that the interactions between the combination of two compounds were additive. More importantly, the combination of three compounds showed a synergetic effect. Although neither lopinavir/ritonavir nor sorafenib used individually resulted in significant inhibition of the parasite when combined the inhibition significant (combination B). Butcher attributed this unexpected result to the combination of two drugs with different biomolecular targets ^47^. Furthermore, the combination of auranofin and lopinavir/ritonavir (combination A) led to a significant reduction in the parasite count compared to amphotericin B possibly because of the strong anti-leishmanial effect of auranofin added to the different mechanism of action exhibit by lopinavir/ritonavir in modulating the immune system. Our findings are supported by Lewis, *et al*. results in an animal model where a combination of auranofin and an antiretroviral drug was able to reduce significantly post-therapy viremia ^48^.

The use of drugs with synergistic or additive activity in combination therapy delays or prevents the development of resistance and may shorten the treatment, which, in turn, decreases the undesirable effects of each drug ^49^^,^^50^. Moreover, this alternative strategy leads to cost and time reductions ^41^. The search for synergism by combining approved drugs can rapidly take to preclinical and clinical phases ^51^.

In an attempt to explore the effect of each drug on the promastigotes, we conducted ultrastructural studies on the treated parasites. SEM of promastigotes treated with auranofin, lopinavir/ritonavir, and sorafenib for 48 hours showed severe shape distortion and loss of flagella with irregularities on the cell surface. Some promastigotes treated with auranofin exhibited a rounded appearance. Sharlow, et al., found the same morphology in *L. amazonensis* promastigotes treated with auranofin ^52^. Rigobello, *et al.*^13^, and Ilari, *et al.*^14^, attributed this rounded swelling to the inhibition of trypanothione reductase and the membrane permeability transition. This morphological distortion had not been observed with any known leishmanicidal drugs ^52^.

In our study, TEM images suggested that auranofin, lopinavir/ritonavir, and sorafenib exerted their anti-leishmanial effect on *L. infantum* promastigotes by inducing apoptosis. There were some changes in the essential organelles including the nucleus, the mitochondria, and the cell membrane in addition to changes in the cytoplasmic contents. There were also irregularities in the cell membrane. The most striking ultrastructure change was the presence of a large number of acidocalcisomes in the cytoplasm, which is important evidence of apoptosis ^53^. Only the promastigotes that were treated with lopinavir/ritonavir showed autophagy in addition to apoptosis. The increased number of vesicles with different densities in the cytoplasm, the rupture of the nuclear envelope, and the presence of dense chromatic granules are signs of autophagy ^24^^,^^54^. The two major forms of programmed cell death, apoptosis and autophagy, were also verified in the ultrastructure study of *L. amazonensis* treated by HIV protease inhibitors ^24^.

In conclusion, administering drug combinations is more effective than that of individual drugs. Drug combinations of two compounds led to additive interactions. Furthermore, those of the three compounds showed synergistic activity. Synergism with this drug combination elicits a structure-function approach in fighting leishmaniasis. The electron microscopic study revealed that the three drugs exerted their anti-leishmanial action by inducing apoptosis, as well as autophagy in the case of lopinavir/ritonavir. The effectivity of this drug combination may be attributed to the similar mechanisms of action of its compounds. However, further experimental studies to establish the curative combination ratio and toxicity parameters of these compounds are needed, as well as others to test drug effectivity on amastigotes.
